# Wide-Field Polarimetric Second-Harmonic Imaging for Rapid and Nondestructive Investigation of Laser-Induced Crystallization Phenomena

**DOI:** 10.1021/acsnano.4c05554

**Published:** 2024-08-23

**Authors:** Seonwoo Lee, Tetsuo Kishi, Yves Bellouard

**Affiliations:** † Galatea Lab, STI IEM, Ecole Polytechnique Fédérale de Lausanne (EPFL), Rue de la Maladière 71b, Neuchâtel CH-2002, Switzerland; ‡ Department of Chemistry and Materials Science, Tokyo Institute of Technology, 2-12-1 Ookayama, Meguro-ku, Tokyo 152-8550, Japan

**Keywords:** wide-field polarimetric second-harmonic microscopy, laser-induced crystallization, nanocrystal-in-glass composites, crystallographic morphologies, chirality

## Abstract

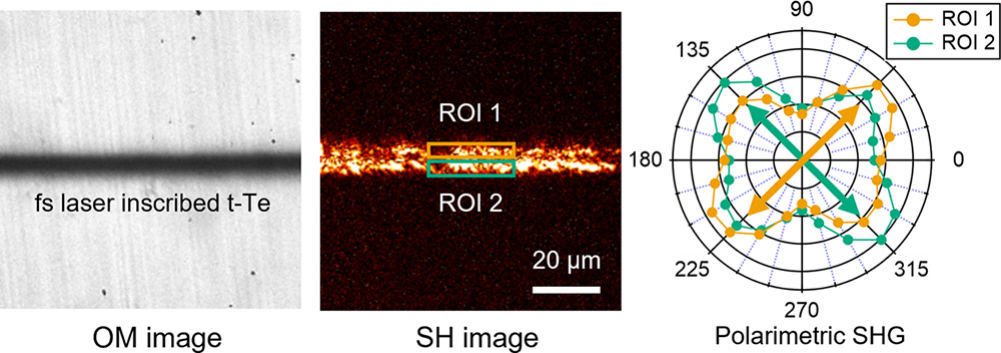

The selective and controlled formation of nanocrystals in glass is emerging as a versatile method to achieve functional photonics, optoelectronics, and quantum devices, such as single-photon emitters. Here, we investigate the use of wide-field polarimetric second-harmonic (SH) microscopy as a method to rapidly and nondestructively examine nanoscale crystal arrangements in laser-processed glass. As a case study, we investigate tellurite glass, where the formation of a trigonal tellurium (t-Te) nanocrystalline phase after femtosecond laser exposure was recently demonstrated. Combined with theoretical models, we show that wide-field polarimetric SH microscopy offers comprehensive information on the nanocrystals’ orientation, distribution, and chirality. With its high imaging throughput and spatial resolution, this method has the potential not only to significantly accelerate investigations on laser-induced glass crystallization processes but also to provide a valuable tool for in situ process monitoring.

## Introduction

Taking roots from ancient, millennia-old experimental chemistry where nanocrystals—in these times not known as such—were mixed in glass to achieve certain colors and artistic effects, nanocrystals embedded in a glass matrix are nowadays emerging as a key technology in the next generation of photonic devices such as liquid crystal displays, light-emitting diodes, lasers, and luminescent solar concentrators.^1,2^

In this context, femtosecond (fs) laser-induced crystallization in glass has received extensive attention recently due to the intrinsic flexibility of the process, allowing for the formation of arbitrary patterns and/or for in-volume nanocrystallization.^3−10^ Peak power density of focused fs laser can reach up to TW cm^–2^, which triggers nonlinear absorption. As this absorption occurs only within the focal volume, the production of crystalline phases can be spatially selective. Using these unique characteristics of fs laser processing, studies have shown the direct writing of electrically conductive channels (e.g., metallization in tellurite-based glass) and the formation of optically active elements (e.g., lithium niobate).^7,11−13^

However, analyzing these crystal nanostructures remains cumbersome due to the small scale of the laser-induced modifications, and it requires destructive investigation means such as transmission electron microscopy (TEM) and associated techniques.^10,14,15^

Finding an in situ nondestructive method to rapidly extract crystal information, as diverse as orientation, location, and distribution, would be highly beneficial not only to accelerate investigations in laser-induced nanocrystallization processes but also to implement in situ monitoring and closed-loop control strategies.

Toward this goal, nondestructive analysis methods, such as Raman^7,16−19^ or Fourier-transform infrared (FTIR) spectroscopy,^20−23^ have been proposed. However, these methods are not ideal for in-line measurements, as they are either too slow for in situ process control or lack the spatial resolution for resolving accurately microscale patterns.

Second-order nonlinear optical microscopy (“second-harmonic (SH) microscopy”) is another promising tool for in situ monitoring of laser-induced crystal structures as it is sensitive to nonisotropic structures, such as crystal orientations or interface arrangements between crystals.^24^

Recently, combined with a fs laser, wide-field SH microscopy demonstrated an improved imaging throughput for BaTiO_3_ by a factor of 5000 over conventional scanning confocal imaging systems.^25^ With this improvement in throughput, wide-field SH microscopy has been widely used in biology to investigate dynamics of biological specimens.^26−32^ However, outside biological studies, wide-field SH microscopy has not received much attention.

Here, we implement wide-field polarimetric SH microscopy as a fast and nondestructive method to comprehensively investigate the crystalline morphologies of femtosecond laser-induced tellurium nanocrystals embedded in tellurite glass (e.g., their spatial distribution, orientation, and chirality).

Recently, our group reported that fs laser irradiation produced elemental trigonal tellurium (t-Te) nanocrystals on tellurite glass surfaces,^18^ through interactions between the ionized material and its surrounding atmosphere. We observed that single-pulse irradiation was sufficient for the formation of the t-Te nanocrystal, and the production of Te was favorable in an open-air atmosphere. We further demonstrated that these nanocrystals can be arranged to form photoconductive patterns.^7^ We also found that the crystallization of t-Te was accompanied by the formation of self-organized nanogratings oriented perpendicular to the inscribed laser polarization.^19^

Using SH signal as a probe, we demonstrate that wide-field SH microscopy enables the visualization of laser-induced crystallization morphologies on a submicrometer scale, within 1 s, while also providing information on spatial distribution, orientation, and chirality. Hence, with its high imaging throughput and comprehensive characterizing capabilities, polarimetric wide-field SH microscopy can efficiently contribute to laser crystallization process implementation by offering means for rapid in situ characterization and monitoring.

## Results and Discussion

### SH Imaging of Laser-Induced t-Te Nanocrystals in Tellurite Glass

**Figure 1:**
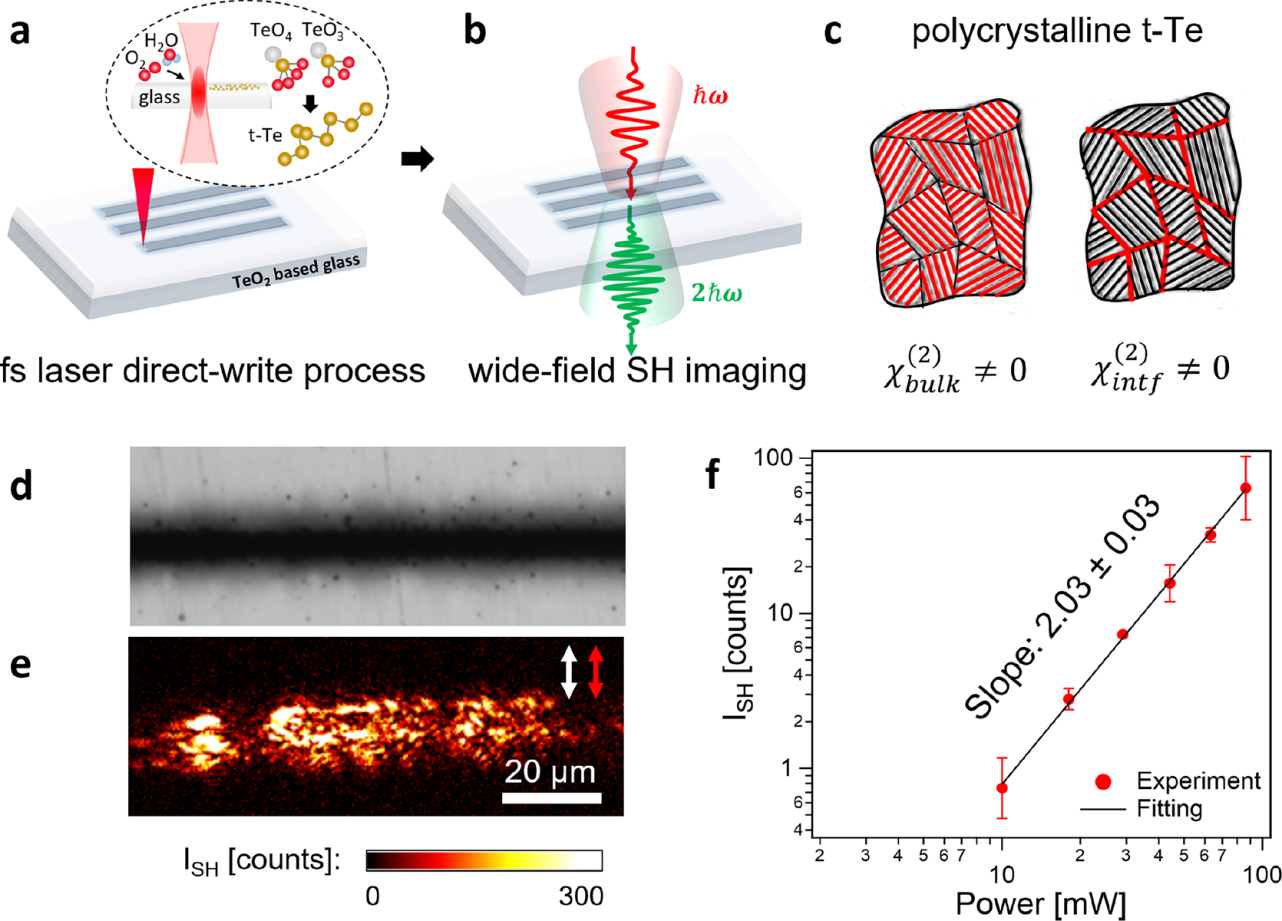
SH imaging of laser-induced t-Te nanocrystals in tellurite glass. Schematic of (a) the fs laser direct writing process and the structural evolution of the tellurite glass upon the laser irradiation and (b) wide-field SH imaging of the laser-induced t-Te. (c) Schematic illustration of polycrystalline t-Te, which can generate SH signals due to two factors. One is the presence of grains with preferential crystal orientations (via a nonzero second-order bulk susceptibility χ_bulk_^(2)^), and the other is the presence of grain boundaries between t-Te nanocrystals (via a nonzero second-order interfacial susceptibility χ_intf_^(2)^). (d) OM image and (e) corresponding SH image of the laser-affected zone on tellurite glass. The laser-inscribed zone is processed at the repetition rate of 1 MHz, the pulse energy of 200 nJ, and the writing speed of 10 mm s^–1^ (incoming pulse fluence: 13 J mm^–2^). The inscribed beam is transversely polarized with respect to the writing direction (white arrow). For the SH image, a probing beam, which is transversely polarized with respect to the writing direction, is used (red arrow). (f) Logarithmic plot of SH intensity of t-Te vs laser power (red dots). The fitted slope (black line) is 2.03 ± 0.03, exhibiting a quadratic increase in the SH response with the incident laser power.

To investigate the laser-induced crystallization of t-Te, we first exposed the surfaces of tellurite glass to a fs laser. Figure 1a shows a schematic of the fs laser direct writing process and the structural evolution of the tellurite glass upon laser irradiation. The fs laser with a high peak power density of ∼ TW cm^–2^ induces nonlinear absorption, which is subsequently followed by avalanche ionization processes.^33^ The ionization leads to molecular bond cleavage, yielding plasma enriched with a high concentration of Te ions. This abundance of Te ions facilitates the formation of Te clusters and eventually the formation of t-Te crystallized nanostructures.^7,18,19^ The formation of t-Te requires an open-air atmosphere containing oxygen and water molecules. The observed depth of the crystallized zone is approximately 350 nm^18^ much shorter than the Rayleigh length of the laser (2.47 μm), indicating that the t-Te formation dominantly occurs on the surface of the tellurite glass.

The femtosecond laser-induced t-Te nanocrystals are then examined using wide-field SH microscopy. The laser-affected zones are illuminated with a loosely focused beam (600 fs, 1 MHz, 1030 nm) at normal incidence, producing a ∼100 μm-diameter illumination area (Figures 1b and S1). Phase-matched SH photons of 515 nm wavelength are consequently emitted in the direction of the surface normal.

Tellurite glass (TeO_2_–WO_3_–K_2_O) does not have long-range organized structures, and hence, no coherent SH photons are generated. In contrast, the laser writing process disrupts the glass network and gives rise to the formation of the polycrystalline t-Te. The t-Te nanocrystals in the laser-affected zone have no longer a random arrangement, allowing for second-harmonic generation (SHG). One of the contributing factors to the SHG is the presence of grains with preferential crystal orientations (i.e., a nonzero second-order “bulk” susceptibility **χ**_bulk_^(2)^), while the second one is the presence of grain boundaries that define interfaces between t-Te nanocrystals (i.e., a nonzero second-order “interfacial” susceptibility **χ**_intf_^(2)^) (Figure 1c).

The total second-order nonlinear polarization in materials ***P***^(2ω)^ can be expressed as a sum of the bulk and interfacial contributions.^34−36^
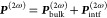
1
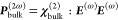
2
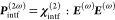
3where **χ**^(2)^s are second-order nonlinear susceptibilities and ***E***^(ω)^ is the incoming electric field.

The coherence length *L*_coh_ for the SHG in the bulk tellurium is expressed by
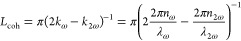
4where *k*_ω_ and *k*_2ω_ are the wavevectors for the fundamental and the SH fields, respectively.^24^ The refractive indexes for tellurium are *n*_ω_ = 4.62 at a wavelength of λ_ω_ = 1030 nm and *n*_2ω_ = 3.08 at λ_2ω_ = 515 nm.^37^ Using eq 4, we obtain the coherence length for bulk tellurium *L*_coh_ = ∼167 nm.

In our previous study, we found that fs laser-induced t-Te nanocrystals form polycrystalline phases, with nanocrystal sizes increasing with the number of irradiated laser pulses.^18^ Under thermally cumulative exposure, an average nanocrystal size of 12.8 ± 2.8 nm has been observed. Since our experiment in this study was conducted using similar experimental conditions in the thermal cumulative regime but involving equal or fewer pulses, we estimate the average nanocrystal sizes to be up to approximately 13 nm. Considering the fact that SHG is a coherent process, the individual SHG signal from t-Te within the calculated coherence length contributes to the total SH response.^38^ Hence, the average nonlinear properties of laser-induced t-Te can indeed be investigated by using SH microscopy.

Figure 1d,e shows the optical microscopy (OM) image and the corresponding SH image of the laser-affected zone on the tellurite glass, respectively. The laser-inscribed zone is processed at the repetition rate of 1 MHz, the pulse energy of 200 nJ, and the writing speed of 10 mm s^–1^ (incoming pulse fluence: 13 J mm^–2^). The inscription beam is transversely polarized with respect to the writing direction. For SH images, a probing beam, which is also transversely polarized with respect to the writing direction, is used. Considering that the unmodified glass has no symmetry, it does not generate an SH response. Hence, we set the average intensity of the unmodified glass as the background level. The SH images are then corrected by the subtraction of the average intensity of the unmodified glass. The acquisition time for each image is 1 s.

The OM and SH images show clear differences. While the OM image exhibits homogeneous optical contrast, the SH image displays heterogeneous contrast, showing a higher intensity at the boundaries of the modified zone compared with the center of the modified zone. This observation is consistent with Raman spectroscopy measurements performed on laser-modified tellurite glass,^18,19^ where the t-Te/TeO_2_ ratio increases at the boundary and decreases toward the center. As noted above, we presume that the SH signal originates not only from the intrinsic bulk crystalline structure but also from the interfaces between crystals. Therefore, this finding implies that the formation of t-Te is more likely to occur at the periphery of the laser-affected zone, where the local temperature is lower than that at the center. Although TeO_2_ can also be present in a crystalline form itself, the resulting crystallinity, if present, may remain low in this case. Consequently, in combination with the results of Raman spectroscopy, we suggest that the SH signal in Figure 1e reports on the localized crystalline area of t-Te.

Furthermore, the SH response of t-Te displays a clear quadratic dependence on the illumination power (Figure 1f). This quadratic dependency indicates that the observed SH signal is caused by the second-order nonlinear optical properties of tellurium.

### Polarimetric Analysis of the SH Response in t-Te Nanocrystals

To further investigate the laser-induced t-Te nanocrystals, we performed SH polarimetric analysis. Polarization-resolved SH measurements provide a way to probe the orientation of crystal structures or the arrangement of interfaces.^24,36^ This is because the magnitude of the second-order bulk/surface susceptibility varies with the polarization direction of the incident light, leading to changes in the SH intensity.

**Figure 2:**
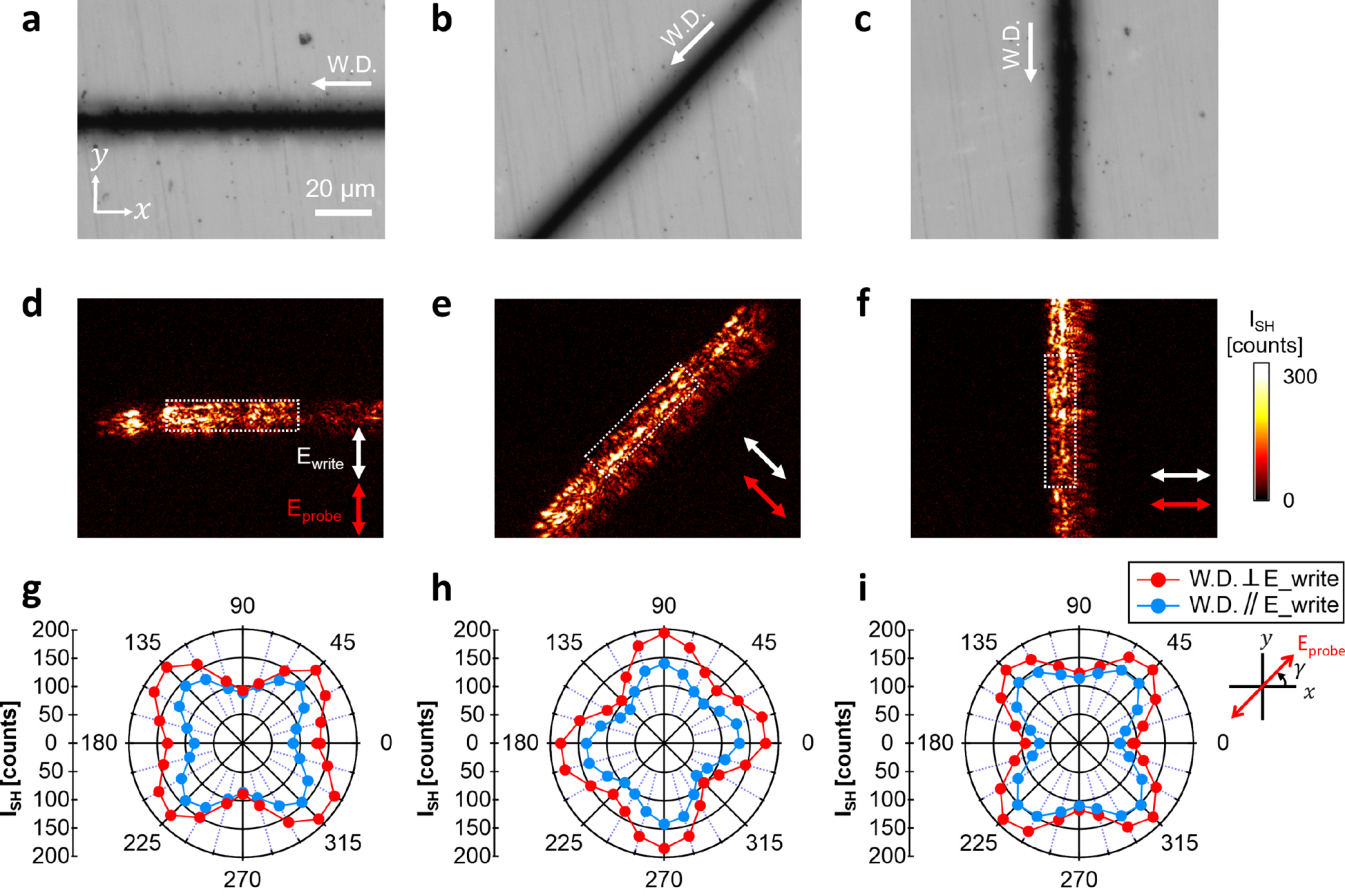
Polarization-resolved SH response in t-Te nanocrystals. (a–c) OM images of the laser-inscribed lines on the tellurite glass in three different writing directions: horizontally (from right to left), diagonally (from top right to bottom left), and vertically (from top to bottom). The lines are written at the repetition rate of 1 MHz, with a pulse energy of 200 nJ and writing speed of 10 mm s^–1^ (incoming pulse fluence: 13 J mm^–2^). (d–f) SH images corresponding to (a–c), measured with transversely polarized light relative to the inscribed line. Insets show the polarization directions for the writing light (*E*_write_) and the probing light (*E*_probe_). (g–i) Polarimetric plots of averaged SH intensity (*I*_SH_) for the horizontal, diagonal, and vertical lines, written by longitudinal (W.D.∥*E*_write_, blue) and transverse (W.D.⊥*E*_write_, red) polarization. SH imaging of laser-affected tracks is performed by changing the polarization direction of the probing beam (*E*_probe_). The angle (γ, inset) between the polarization direction of the incident beam and the *x*-axis varies from 0 to 360° with an interval of 15°.

Figure 2a–c shows the OM images of the laser-inscribed lines on the tellurite glass. We inscribe laser patterns in three different writing directions (W.D.): horizontally (from right to left), diagonally (from top right to bottom left), and vertically (from top to bottom). The lines are written at a repetition rate of 1 MHz, with a pulse energy of 200 nJ and a writing speed of 10 mm s^–1^ (incoming pulse fluence: 13 J mm^–2^). For a given writing direction, two different writing polarization (*E*_write_) states are used: longitudinally (W.D.∥*E*_write_) or transversely (W.D.⊥*E*_write_) polarized light. Figure 2a–c displays the case inscribed with transverse polarization.

Next, we conducted wide-field SH imaging of laser-inscribed tracks by changing the polarization direction of the probing beam (*E*_probe_). The angle (γ) between the polarization of the probing beam and the *x*-axis varies from 0 to 360° with an interval of 15°. In Figure 2d–f, we show the SH images corresponding to Figure 2a–c, measured with transversely polarized light relative to the inscribed line. In Figure 2g–i, we display the polarimetric plots of the averaged SH intensity (*I*_SH_) for the horizontal, diagonal, and vertical lines, written by longitudinal (blue) and transverse (red) polarization. The region of interest (ROI), where SH intensity is obtained, is indicated by the white dotted box in Figure 2d–f.

We observe that SH signals for t-Te are not uniformly distributed, regardless of the laser writing direction (Figure 2d–f). A higher intensity is detected at the boundaries compared to the center for all the modified lines. The SH intensity profiles perpendicular to the laser writing direction for these modified lines are shown in Figure S2. This observation shows that the crystal morphology is dominantly affected by the local variation in laser fluence, with a higher fluence at the center of the beam compared to the boundaries.

Furthermore, Figure 2g–i shows that the averaged SH intensity of the modified zone varies with the direction of the probing polarization. For example, in Figure 2g, the horizontal line displays the strongest intensity at γ = 45 and 315°.

As noted above, the SH polarimetric plots reflect the orientation of the bulk (e.g., crystallized grain) or the orientation of the interfaces (e.g., grain boundaries). It has been found from transmission electron microscopy that laser-induced t-Te has polycrystalline nature (i.e., each grain with a different orientation).^18^

In the case of a polycrystalline structure lacking a preferential orientation among crystalline grains and boundaries, the same SH intensity is expected for any incoming polarization. However, the horizontal line in Figure 2g exhibits the strongest intensity at γ = 45 and 315°. In other words, when the polarization direction of the incident beam is rotated by γ = ±45° relative to the writing direction, the strongest intensity is observed. Similarly, the strongest peaks are observed at γ = 90 and 0° in Figure 2h and at γ = 135 and 45° in Figure 2i. This observation suggests that laser-induced t-Te possesses a nonrandom crystalline texture with a preferential arrangement.

Moreover, we observe a higher SH intensity for the line inscribed with transverse polarization (W.D.⊥*E*_write_, red) compared to longitudinal polarization (W.D.∥*E*_write_, blue). Considering that the SH signal originates from t-Te, the difference in SH intensity suggests a higher amount of t-Te in the lines written with transverse polarization compared to those written with longitudinal polarization. Previous studies have revealed that laser-induced t-Te is accompanied by the formation of self-organized periodic nanogratings perpendicular to the laser polarization.^19^ In other words, laser writing with transverse polarization creates nanogratings oriented horizontally to the writing direction, whereas that with longitudinal polarization creates nanogratings oriented vertically. Therefore, the observed discrepancy in SH intensity implies that the polarization direction or the nanograting orientation influences the degree of crystallization.

Interestingly, despite the difference in SH intensity, the SH polarimetry peaks are observed at the same angle. As a result, this finding indicates that both the inscribing polarization direction and the organization of the nanograting do not affect the crystalline arrangement.

### Local Polarimetric Analysis of the SH Response in t-Te Nanocrystals

**Figure 3:**
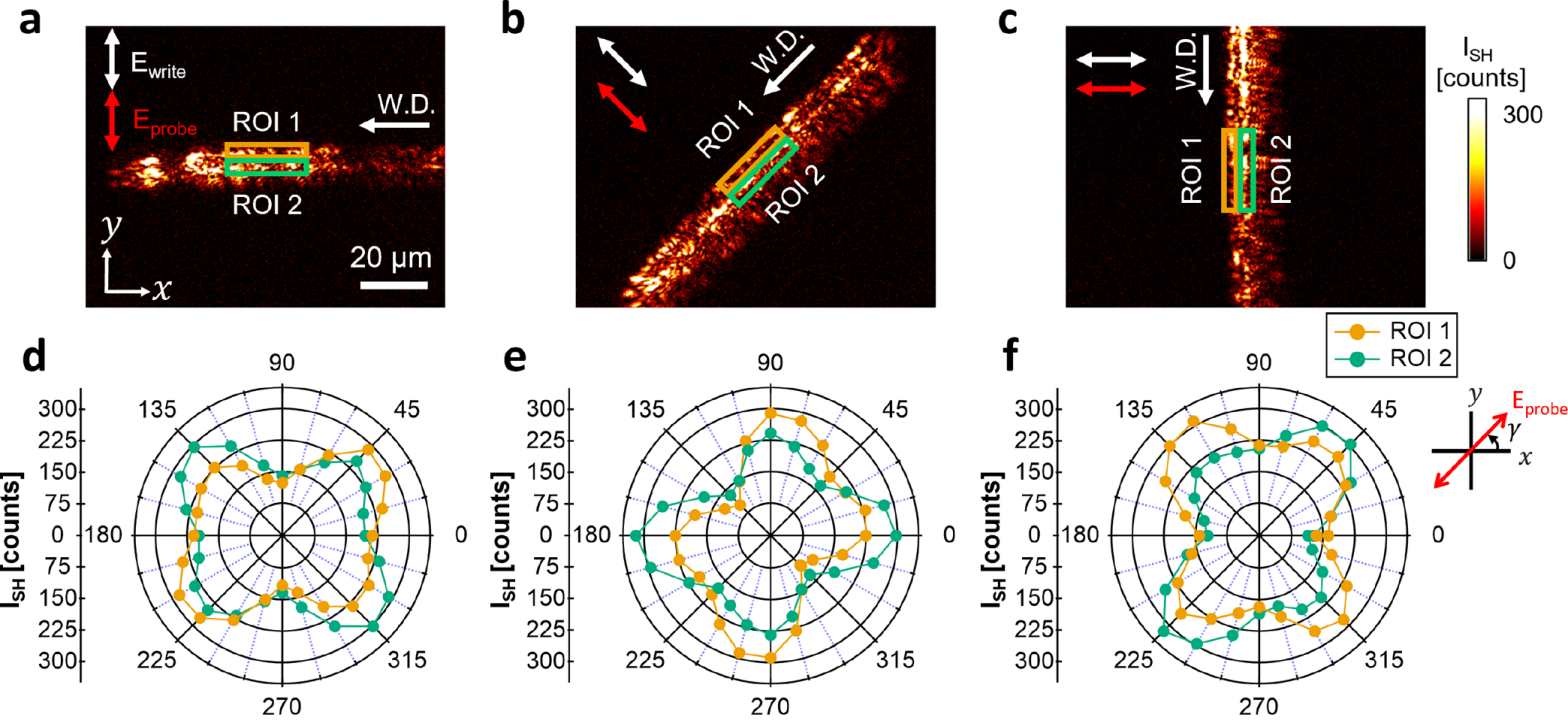
Localized polarimetric SH response in t-Te nanocrystals. (a–c) SH images of laser-inscribed lines on the tellurite glass taken from Figure 2d–f, with an indication of two distinct regions of interest (ROI 1 and ROI 2) used for localized polarimetry analysis. (d–f) Localized SH polarimetric results for the horizontal, diagonal, and vertical lines, inscribed by transversely polarized light. The polarimetry plots for ROI 1 (orange) and ROI 2 (green) display a similar degree of inclination yet in opposite directions with respect to the laser writing direction.

To obtain a deeper understanding of the spatial arrangement of laser-induced t-Te, we further investigate the SH polarimetry within a localized area. We set two distinct regions of interest (ROIs) with opposite sides of the inscribed lines for localized polarimetry analysis, as shown in Figure 3a–c.

Figure 3d–f shows the SH polarimetry results for the horizontal, diagonal, and vertical lines, written by transversely polarized light. We observe different polarimetry plots for ROI 1 (orange) and ROI 2 (green), exhibiting a similar degree of inclination, yet in opposite directions with respect to the laser writing direction.

Figure 3d, for example, shows that the maximum SH intensity for ROI 1 is at γ = 45° and that for ROI 2 is at γ = 315°. In other words, the strongest intensity is observed at angles of +45° in ROI 1 and −45° in ROI 2 with respect to the writing directions.

Similarly, the diagonal line, as shown in Figure 3e, displays the maximum SH intensities for ROI 1 and ROI 2 at γ = 90 and 0°. The vertical line has the maximum SH intensities for ROI 1 and ROI 2 at γ = 135 and 45°, respectively. Thus, this observation suggests that the crystalline morphologies on two halves of the line are symmetrically arranged relative to the writing direction.

Moreover, we obtain a different polarimetric plot for the two halves of the line inscribed by the same linear polarization. We thus presume that the structural arrangement of t-Te is independent of one of the nanogratings. In the following, we quantify the t-Te crystalline arrangement using theoretical models.

### Theoretical Calculation of the SHG from t-Te Nanocrystals

**Figure 4:**
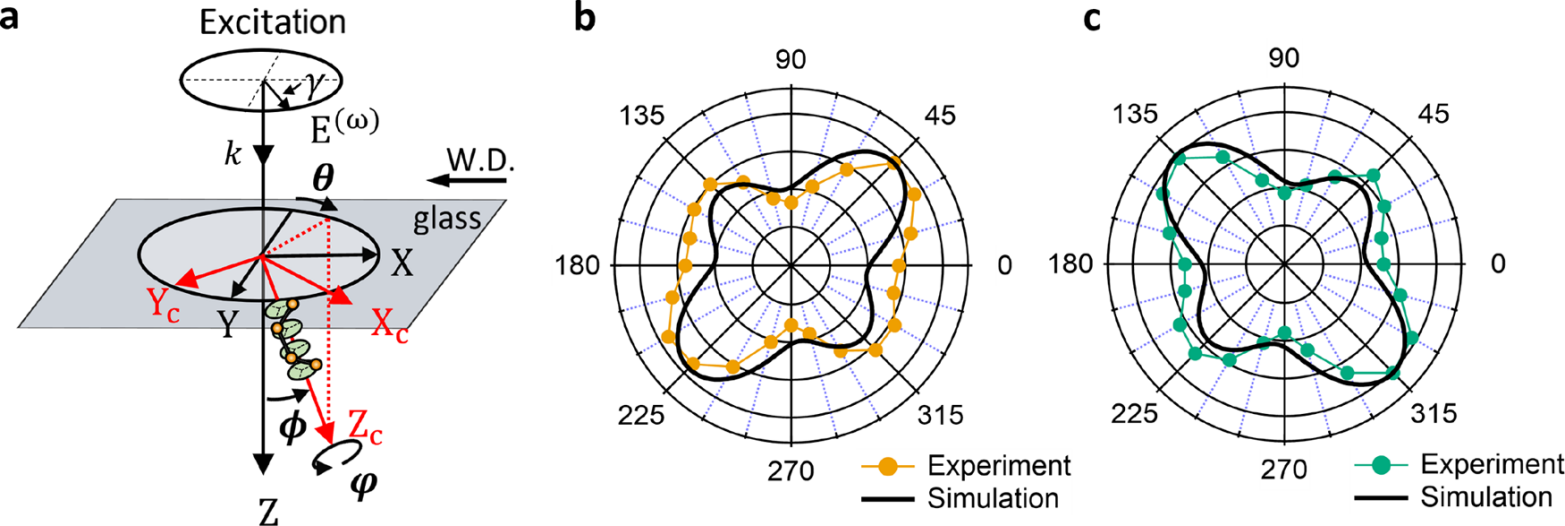
Theoretical calculation of the SH response of t-Te under a linearly polarized beam. (a) Schematic diagram of a t-Te nanocrystal, where the orientation of the *c*-axis (*Z*_*c*_ axis) is determined by θ, ϕ, and φ. The writing direction is aligned parallel to the *X*-axis. The incident beam propagates along the *Z*-axis, and the polarization angle γ of the beam is defined relative to the *X*-axis. (b) Simulated polarimetric SH response at θ = 135 or 315° with ϕ = 10°, φ = 0°, together with the experimental result (ROI 1 in Figure 3d). (c) Simulated polarimetric SH response at θ = 45 or 225° with ϕ = 10°, φ = 0°, along with the experimental result (ROI 2 in Figure 3d). Experimental data are represented by dots (orange and green), while theoretical data are shown by lines (black).

The crystal structure of t-Te consists of helical chains arranged in a hexagonal array, as depicted in Figure 4a.^39,40^ It is classified into space groups, either *P*3_2_21 (*D*_3_^6^) or *P*3_1_21 (*D*_3_^4^), depending on whether it exhibits a left-handed or right-handed helix configuration.

In the space group of *D*_3_^6^ or *D*_3_^4^, there are five nonzero elements in the second-order susceptibility tensor with two independent elements being χ_*xxx*_^(2)^ = −χ_*xyy*_^(2)^ = −χ_*yxy*_^(2)^, and χ_*xyz*_^(2)^ = −χ_*yzx*_^(2)^. It has been found that the χ_*xxx*_^(2)^ element is equal for both helical structures (*D*_3_^6^ and *D*_3_^4^), but the sign of the χ_*xyz*_^(2)^ element is different.^40^ As both structures exhibit mirrored characteristics, we present the simulation results only for space group *D*_3_^6^ in the following discussion. Based on the results for space group *D*_3_^6^, we can infer those for space group *D*_3_^4^.

The orientation of t-Te in the three-dimensional (3D) space can be described using three Euler angles. Specifically, the orientation of the *c*-axis of the t-Te nanocrystal can be characterized by the angles θ, ϕ, and φ in spherical coordinates, as illustrated in Figure 4a. To simulate the SH response of t-Te for given θ, ϕ, and φ, we assume that the shape of the t-Te nanocrystal is spherical and that its size is much smaller than the wavelength of the beam. We also assume that the incident beam propagates along the *Z*-axis and the excitation polarization angle γ can be adjusted in the *XY* plane by a half-wave plate.

The excitation field ***E***^(ω)^ is decomposed into three orthogonal components along the three axes in the lab coordinate frame, i.e., ***E***^(ω)^ = *E*_*x*_*e*_*x*_ + *E*_*y*_*e*_*y*_ + *E*_*z*_*e*_*z*_, where *e*_*x*_, *e*_*y*_, and *e*_*z*_ are unit vectors, as shown in Figure 4a. Depending on the angles θ, ϕ, and φ, the electric field in the crystal frame ***E***_c_^(ω)^, where ***E***_c_^(ω)^ = *E*_c*x*_*e*_c*x*_ + *E*_c*y*_*e*_c*y*_ + *E*_c*z*_*e*_c*z*_, changes and is related to ***E***^(ω)^ by
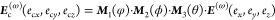
5
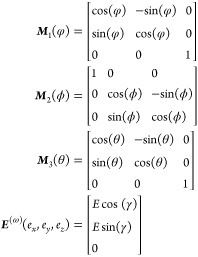
6SH polarization ***P***_c_^(2ω)^ in the crystal frame is given by
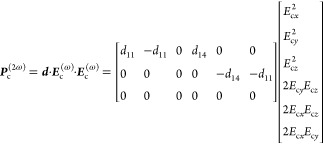
7where ***d*** is the second-order susceptibility tensor for t-Te. In simulation, the following values are used: *d*_11_ = 1840 pm V^–1^ and *d*_14_ = −1200 pm V^–1^.^40,41^

As we assume that the size of the nanocrystal is much smaller than the wavelength, the SHG polarization within the nanoparticle can be considered three orthogonal SHG dipoles along the crystal axes. As a result, the total SHG intensity *I*_SH_ can be expressed as
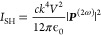
8where *c* is the speed of light, *k* is the wavenumber at SH frequency, *V* is the volume of the nanocrystals, and ϵ_0_ is the vacuum permittivity.^42^

Using eqs 5–8, we simulate the SH response emitted from t-Te for various orientations of the nanocrystal and the excitation polarizations. Figure S3 displays the evolution of polarization-dependent SHG responses of t-Te as a function of θ, with fixed values of ϕ = 10° and φ = 0°. Notably, as the projection of the *c*-axis of t-Te on the *XY* plane rotates (θ changes), the cross-shaped polar plot rotates, showing the same plot every 180°.

When the t-Te nanocrystal is oriented at either θ = 135 or 315°, with ϕ = 10° and φ = 0°, we find a good agreement between simulated (solid black line) and experimental results (orange dots) obtained from ROI 1 in Figure 3d, as shown in Figure 4b. Similarly, the simulated result matches with the experimental result (green dots) obtained from ROI 2 in Figure 3d when the t-Te is oriented at either θ = 45 or 225°, with ϕ = 10° and φ = 0° (Figure 4c). Considering that θ is defined in the *XY* plane, this finding suggests that on average, the *c*-axis of t-Te nanocrystals on ROI 1 and ROI 2 in Figure 3a is symmetrically arranged at ±45 or ±225° with respect to the laser writing directions (*X*-axis). Note that the calculated orientation of the *c*-axis represents the averaged characteristics of t-Te nanocrystals as they have a polycrystalline structure.

In addition, the simulation results exhibit that the c-axes in ROI 1 and ROI 2 are directed toward the surface normal, with a slight tilt of ϕ = 10° from the *Z*-axis. Previous studies have shown that an open-air atmosphere containing oxygen and water molecules is necessary for the formation of laser-induced t-Te.^18^ Therefore, we presume that the production of t-Te unfolds from the surface to the bulk, thereby inducing the orientation of the *c*-axis toward the surface normal.

Furthermore, since t-Te consists of 3-fold-symmetric helical chains, the SH response periodically changes every 120° as the *c*-axis of t-Te rotates around its axis (φ changes), as shown in Figure S4.

Because of the mirrored characteristics, we also obtain identical simulation results for both left-handed and right-handed configurations when their c-axes are symmetrically positioned relative to the origin.

As a result, we reveal that despite its polycrystalline nature, laser-induced t-Te nanocrystals exhibit a nonrandom arrangement. In the sequel, based on the experimental and simulated results, we propose the crystallization mechanism of t-Te produced by fs laser irradiation.

### Proposed Crystallization Mechanism of t-Te Produced by fs Laser Irradiation

**Figure 5:**
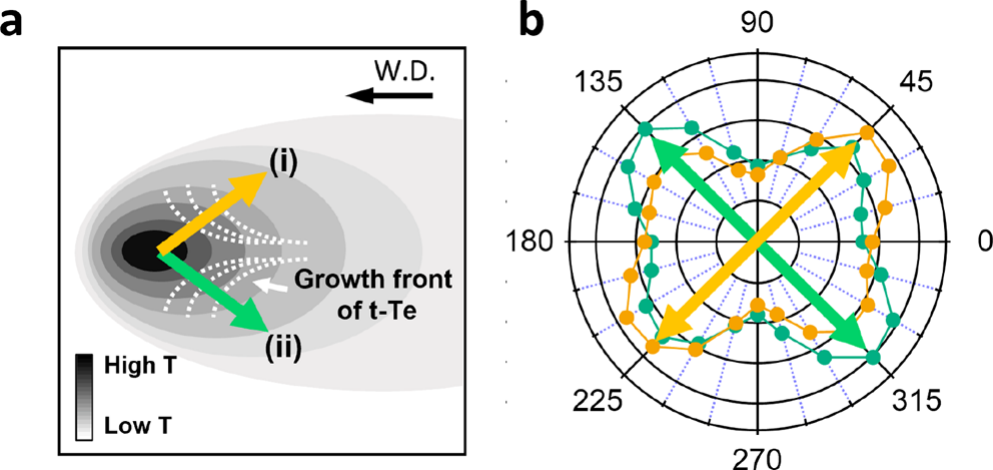
Potential crystallization mechanism of t-Te produced by fs laser irradiation. (a) Contour plot of the temperature distribution as the laser beam is scanned from right to left on tellurite glass. Due to the delayed nucleation in the center caused by the high temperature, the crystallization starts from the periphery than the center. This results in growth front lines (white dotted lines), which are not parallel relative to the writing direction. These bouquet-like growth fronts give rise to different SH responses in different local areas due to their distinct crystal orientations. (b) Localized SH polarimetric plots for the horizontal line from Figure 3d. On the upper side of the writing line (i, orange), the SH signal is enhanced when the polarization is tilted at an angle of 45°. Conversely, in the lower side of the line (ii, green), the SH signal is increased when the polarization is tilted at an angle of 315°.

The potential crystallization mechanism is schematically depicted in Figure 5a.

#### Step 1. Nonlinear Absorption, Ionization, and Heat Transfer to the Tellurite Glass

First, the fs laser with a high peak power density of TW cm^–2^ induces nonlinear absorption, which is subsequently followed by avalanche ionization processes.^33^ The ionization results in molecular bond cleavage, yielding plasma enriched with a high concentration of Te ions.

Simultaneously, due to carrier–carrier scattering or carrier–phonon scattering, thermalization occurs, and consequently, a temperature gradient arises on the surface of the specimen. It is known that the temperature gradient is distributed over a distance of several laser beam diameters.^43^Figure 5a shows a contour plot of the temperature distribution as the laser beam is scanned from right to left on the tellurite glass. The temperature of the glass reaches the maximum value at the center of the laser trace and decreases away from the center.

#### Step 2. Nucleation

As the beam moves, the irradiated area undergoes a rise and a subsequent fall in temperature. When the temperature cools, the glass undergoes structural relaxation, transitioning from amorphous tellurite to a polycrystalline tellurium state. When the temperature falls below the melting temperature (*T*_m_), the formation of seeds (i.e., nucleation) takes place.^44^ Two different nucleation mechanisms may possibly occur: homogeneous (via thermally induced chemical composition fluctuations) and heterogeneous nucleation (via structural defects).^45^ Such nucleation gives rise to the formation of molecular aggregates (e.g., Te_2_ or Te_n_ clusters). It should be noted that the temperature is higher in the center than the periphery; therefore, it takes more time to be quenched to reach *T*_m_ in the center, resulting in a delayed nucleation in this region.

#### Step 3. Crystal Growth of t-Te

Crystal growth of t-Te occurs along the scanning direction, starting from the nucleation site and progressing opposite the laser scanning direction. Due to the delayed nucleation in the center caused by the higher temperature, the crystallization starts from the periphery rather than from the center.

#### Step 4. Formation of the Bouquet-Like Structure

Since the crystallization in the center is constrained by the delayed nucleation and since the laser is scanned over the surface, the resulting structures exhibit a bouquet-like pattern, as shown in Figure 5a (white dotted line). Consequently, the growth front lines are not parallel relative to the writing direction, and the bouquet opens in the opposite direction of the writing direction. Hence, one half side of the line experiences crystal growth inclined relative to the writing direction, while the other half side exhibits the opposite inclination. Such a symmetric configuration is expected from the theoretical calculations in Figure 4. Note that this bouquet-like configuration also has been observed when lithium niobium silicate (LNS) becomes crystallized under fs laser exposure.^46,47^ We further tested for the possible presence of a chevron-shaped stress distribution as reported in Gecevičius et al.^48^ The cross-polarized images of laser-modified tellurite glass showed negligible optical contrast, suggesting no detectable laser-induced birefringence (Figure S5). Thus, it is unlikely that stress induced by a possible chevron-like structure is responsible for the cross-shaped polarimetric SH plot.

Consequently, these bouquet-like configurations give rise to different polarimetric SH responses in different local areas due to their distinct crystalline orientations. As depicted in Figure 5a,b (i, orange), on the upper side of the writing line, the enhanced SH signal is observed when the polarization is tilted at an angle of 45°. Conversely, on the lower side of the line (ii, green), the increased SH signal is observed when the polarization is tilted at an angle of 315°. This interpretation aligns with the results of the localized SH polarimetry in Figure 3d.

Up to now, we have examined the t-Te crystalline structure by using linearly polarized light. In the last section, we used circularly polarized light to investigate the circular dichroism of the SHG for t-Te.

### Circular Dichroism of the SHG for Laser-Induced t-Te

As noted above, t-Te has a chiral structure with either left-handed or right-handed helical configurations. The chiral structure is known to exhibit circular dichroism of the SHG (SHG-CD), exhibiting different SH responses under left-handed circularly polarized light (LCP) and right-handed circularly polarized light (RCP).^49,50^

To examine the chirality (or handedness) of laser-induced t-Te, we next performed a SHG circular dichroism measurement. Different laser parameters, such as writing directions, writing polarizations, and writing speeds, are used for this investigation. To induce circular polarization, a quarter-wave plate is added after the half-wave plate. By rotation of the quarter-wave plate, the handedness of circular polarization is altered.

**Figure 6:**
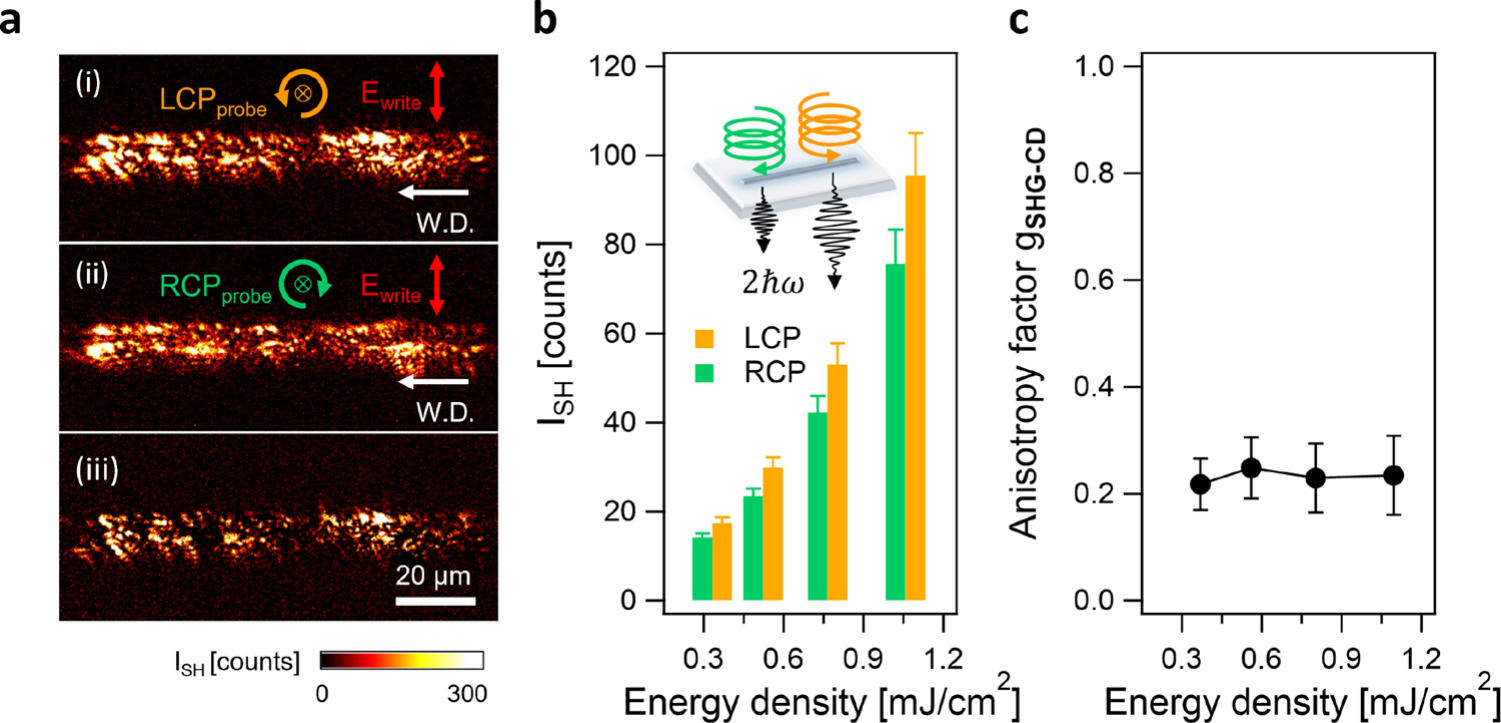
Second-order nonlinear circular dichroism in the laser-induced t-Te. (a) SH images of the laser-inscribed line on the tellurite glass under (i) left-handed circularly polarized light (LCP) and (ii) right-handed circularly polarized light (RCP), together with (iii) subtracted SH image between (i) and (ii). The laser-inscribed zone is processed using the same experimental condition as in Figure 2d. (b) Averaged SH intensity for the t-Te tracks in (a) under the LCP (orange) and RCP (green) light as a function of illumination energy density ranging from 0.35 to 1.10 mJ cm^–2^. A higher intensity is observed under LCP light than RCP light. (c) Extracted SHG anisotropy factor *g*_SHG-CD_ from (b).

We first investigated the effect of writing directions on SHG-CD of t-Te. Figure 6a depicts the SH images of the line measured with (i) LCP and (ii) RCP light. The lines are written from right to left at the repetition rate of 1 MHz, with the pulse energy of 200 nJ and the writing speed of 10 mm s^–1^. The SH images are obtained with different illumination energy densities ranging from 0.35 to 1.10 mJ cm^–2^. The results for the lines written in opposite directions (from left to right) are shown in Figure S6.

For both writing directions, a higher SH signal is observed for LCP compared to that for RCP (Figures 6b and S6b). This finding indicates that laser-induced t-Te comprises an unequal mixture of left-handed and right-handed helical configurations that are oriented out of the imaging plane, thereby leading to the circular dichroism of the SHG.

To quantify SHG-CD for these lines, we use the SHG anisotropy factor *g*_SHG-CD_, defined as^49,50^
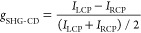
9where *I*_LCP_ and *I*_RCP_ are the SH intensities excited by the left- and right-circularly polarized light, respectively.

We obtain similar values of *g*_SHG-CD_ = ∼0.25 regardless of the writing directions (Figures 6c and S6c). In addition, changes in illumination energy density do not affect the values of *g*_SHG-CD_. As a result, we reveal that laser-induced t-Te nanocrystals consist of unequal amounts of left and right helix configurations. This ratio remains unaffected by the laser writing direction and the incoming power density.

Moreover, the subtracted image between those measured with (i) LCP and with (ii) RCP displays an increase in SH contrast at the edge of the laser-affected zone (Figures 6a(iii) and S6a(iii)).

We next investigated the impact of writing polarization on the SHG-CD of t-Te (Figure S7). Similar to the SH results under linearly polarized light (Figure 2g–i), the lines inscribed with transversely polarized light (W.D.⊥*E*_write_) demonstrate a higher SH intensity compared to those written with longitudinally polarized light (W.D.∥*E*_write_). Both lines exhibit a greater SH intensity when excited by LCP rather than RCP. Furthermore, as illustrated in Figure S7c, a slightly higher anisotropy factor is exhibited for lines written by transverse polarization than for those written by longitudinal polarization.

Hence, the results in Figure S7 show that the anisotropy factor increases with the rise in SH intensity. Considering that SH intensity is proportional to the amount of t-Te, this finding suggests that the anisotropy of chiral configurations becomes more pronounced as more t-Te is produced by the laser.

Lastly, we study the effect of the writing speed on SHG-CD of t-Te. The writing speed *v* is related to the net fluence *E*_deposited_ by *E*_deposited_ = (4*E*_p_/π*w*)(*f*/*v*), where *E*_p_ is the pulse energy, *w* is the optical beam waist, and *f* is the laser repetition rate. To achieve different net fluences, the laser-inscribed lines are written with the same pulse energy of 200 nJ and the same repetition rate of 1 MHz, but we vary the writing speeds ranging from 0.5 to 12.5 mm s^–1^, resulting in different net fluences ranging from 262 to 10.5 J mm^–2^.

**Figure 7:**
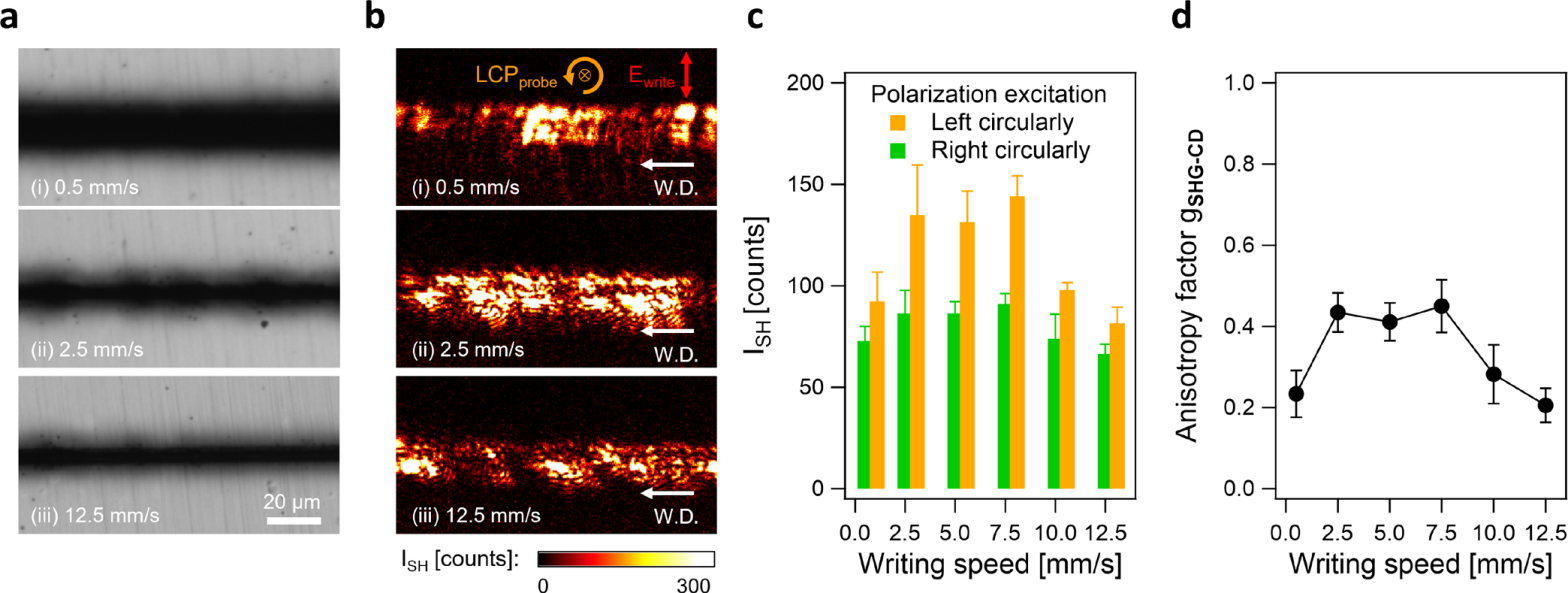
Effect of writing speeds on the SHG-CD of fs laser-induced t-Te. (a) OM images and (b) corresponding SH images of the laser-inscribed lines, written with the same pulse energy of 200 nJ and the same repetition rate of 1 MHz, the same transverse polarization, but different writing speeds of (i) 0.5 mm s^–1^, (ii) 2.5 mm s^–1^, and (iii) 12.5 mm s^–1^. (c) Averaged SH intensity of the laser-inscribed lines measured under LCP (orange) and RCP (green) light at different writing speeds, ranging from 0.5 to 12.5 mm s^–1^, resulting in different net fluence ranging from 262 to 10.5 J mm^–2^, respectively. (d) SHG anisotropy factor *g*_SHG-CD_ of t-Te produced by different writing speeds.

Figure 7a,b shows the OM images and the corresponding SH images of lines inscribed at writing speeds of (i) 0.5 mm s^–1^, (ii) 2.5 mm s^–1^, and (iii) 12.5 mm s^–1^. In the OM images, the width of the laser-affected zone expands as the writing speed decreases. The expansion is caused by the higher net fluence, leading to increased heat accumulation and diffusion over larger areas.

Figure 7c exhibits the averaged SH intensity as a function of the writing speeds under LCP and RCP light. The averaged SH intensity increases as the speed rises from 0.5 to 2.5 mm s^–1^, followed by the gradual decrease in the SH intensity for speeds higher than 7.5 mm s^–1^. These results suggest that the amount of laser-induced t-Te varies with the writing speeds.

We attribute these results to two factors. First, the thermal gradient along the laser writing path is influenced by the speed of writing. The second is the discrepancy between the speed of the crystal growth front and that of writing.

As the writing speed increases (e.g., from *v* = 0.5 mm s^–1^ to *v* = 2.5 mm s^–1^), a thermal gradient increases along the laser writing direction. The increased thermal gradient leads to the directional crystal growth, enhancing the anisotropy of crystalline morphology and thus increasing SH intensity.

At higher scanning speeds (e.g., *v* = 12.5 mm s^–1^), the growth front fails to keep up with the scanning speed. Consequently, crystal growth tapers off and eventually lags behind, inhibiting further growth. This discrepancy between the speed of the crystal growth front and that of writing yields less effective crystallization, consequently reducing the SH intensity. These findings suggest that the degree of crystallization can be controlled by adjusting the writing speeds.

Additionally, the anisotropy factor varies with writing speeds (Figure 7d), reaching *g*_SHG-CD_ = 0.45 at 7.5 mm s^–1^. Note that *g*_SHG-CD_ increases as the SH intensity rises. This correlation between the intensity and anisotropy demonstrates the preferred handedness of t-Te produced by the laser.

The reason for this anisotropy in the helical configuration is beyond the scope of this study. Nonetheless, SH microscopy provides insights into which laser parameters influence the circular dichroism of the produced t-Te. Additionally, the simulation results in Figure 4 explain why the strong SHG-CD signal of t-Te is detected in our experiment: the *c*-axis is almost parallel to the direction of the light propagation. Due to this alignment out of the imaging plane, t-Te can interact differently with LCP and RCP light, translating into the anisotropy factor.

## Conclusions

In summary, we demonstrate a nondestructive and highly sensitive polarimetric wide-field SH microscopy method that we applied to the case of femtosecond laser-induced crystallization.

As an illustration of the method, we observe nonuniformly distributed t-Te nanocrystals along the laser-inscribed tracks. Using SH polarimetry measurements together with theoretical calculations, we find that laser-induced t-Te exhibits a directional arrangement, showing a nonrandom crystalline texture. Specifically, the *c*-axes of t-Te nanocrystals on opposite sides of inscribed lines are symmetrically inclined at ±45 or ±225° with respect to the laser writing directions. Interestingly, the orientation of inscribing laser polarization has a negligible impact on the crystalline morphology. Based on our findings, we propose a potential mechanism for laser-induced t-Te crystallization.

Furthermore, we examine the chirality of the t-Te using the circular dichroism of the SHG measurement. We reveal that laser-induced t-Te nanocrystals comprise unequal amounts of left and right helical configurations. We observe distinct effects of different laser parameters, such as writing directions, writing polarizations, and writing speeds, on the SHG anisotropy factor, reaching up to *g*_SHG-CD_ = 0.45.

The method is generic and potentially applicable to a large number of situations involving nanocrystals embedded in transparent substrates, including nanocrystal-in-glass composites. With its high imaging throughput and comprehensive characterizing capability, we believe that wide-field polarimetric SH microscopy will contribute not only to accelerating laser crystallization processes by providing means for rapid in situ monitoring but also to the broader field of “nanocrystal in transparent media” (including nanocystal-in-glass composites) by providing a rapid and nondestructive method to retrieve key nanocrystal information, such as texture and morphology.

## Materials and Methods

### Glass Specimen Preparation

TeO_2_–WO_3_–K_2_O (TWK) glass systems were used in this study. The composition of TWK glass was 80 TeO_2_–10 WO_3_–10 K_2_O in mol %. To produce TWK glass systems, commercial powders of K_2_CO_3_ (99.5%), WO_3_ (99%), and TeO_2_ (99%) were mixed and melted in an Au crucible at 973 K for 30 min in an electric furnace. The melt was cooled on a brass plate. Subsequently, the glass specimen was crushed, remelted at 973 K for 30 min, and annealed at 598 K for 1 h. The specimen was cut and polished before femtosecond laser inscriptions.

### Femtosecond Laser Machining

A Yb fiber-amplifier femtosecond laser (270 fs pulses, 1030 nm wavelength, 1 MHz, Yuzu, Amplitude) was used to inscribe laser patterns. The specimen was translated under a laser focus using a high-precision motorized stage (Ultra-HR from PI Micos). The laser beam was focused on the surface of the specimen with a 0.4 numerical aperture (NA) objective (OFR-20× -1064 nm from Thorlabs), resulting in a spot size (defined at 1/*e*^2^) of 1.94 μm. The repetition rate was fixed at 1 MHz, which lies well within the heat cumulative regime for TWK glass.^18^ The laser power was set to 200 mW. Laser patterns were lines with a length of 150 μm on the surface of TWK glass. The lines were written by three different directions (i.e., horizontal, vertical, and diagonal) of laser beam movement and two different linear polarization states defined as parallel and perpendicular to the writing direction.

### Second-Harmonic Imaging

Wide-field SH imaging was performed by using a diode-pumped femtosecond laser (600 fs pulses, 1030 nm wavelength, 1 MHz, Yuja from Amplitude SA), as illustrated in Figure S1. A zero-order λ/2 wave plate (WPH05M-1030, Thorlabs) and a polarizing beam splitter (PBS205, Thorlabs) were used to attenuate the intensity of the laser beam. The laser beam was expanded to fill the entrance pupil of the focusing objective lens (Thorlabs MicroSpot LMH-20X-1064, NA = 0.40). The polarization of the beam was controlled with a zero-order λ/2 wave plate (WPH05M-1030, Thorlabs) for linear polarization or a zero-order λ/4 wave plate (WPQ05M-1030, Thorlabs) for circular polarization. The beam was loosely focused on the specimen using the objective lens to create a 100 μm-diameter illumination area. The laser average power was set to 85 mW. High-precision motorized stages were used to move the specimen within a plane perpendicular to the optical axis, while the focusing objective was attached to a third stage translating along the optical axis. The phase-matched SH photons from the specimen were collected by a customized infinity-corrected, collection objective lens. This objective lens was developed based on an arrangement of off-the-shelf fused silica lenses. To block the fundamental beam, potential two-photon excited fluorescence (2PF), and third harmonic generation (THG), three consecutive narrow bandpass SHG filters (Edmund Optics Ltd., BP 515 ± 5 nm OD4 ⌀ 25.0 mm) were utilized. A 10 cm tube lens (LA4380-UV, Thorlabs) focused the SH beam onto the CMOS low-noise imager (C15550–20UP, Hamamatsu photonics). All SH images were measured with the acquisition time of 1 s.

## Supporting Information Available

SH imaging technique for fs laser-induced t-Te nanocrystals on tellurite glass, SH intensity profile perpendicular to the laser writing direction for horizontally, diagonally, and vertically modified lines, theoretical calculation of the θ-dependent polarimetric SH response of t-Te, theoretical calculation of the φ-dependent polarimetric SH response of t-Te, cross-polarized images of the fs laser-inscribed tracks on tellurite glass, effect of writing directions on the SHG-CD of fs laser-induced t-Te, and effect of writing polarization directions on the SHG-CD of fs laser-induced t-Te (PDF)
