# Supplementary material for: Wide-Field Polarimetric Second-Harmonic Imaging for Rapid and Nondestructive Investigation of Laser-Induced Crystallization Phenomena
Source: ACS Nano. 2024 Aug 23;18(36):24929–40. doi: 10.1021/acsnano.4c05554 (PMC11394348; doi:10.1021/acsnano.4c05554)
Supplement: Supplementary file 1 — nn4c05554_si_001.pdf [file nn4c05554_si_001.pdf]

Supporting Information: Wide-Field Polarimetric Second-Harmonic Imaging for Rapid and Nondestructive Investigation of Laser-Induced Crystallization Phenomena

Seonwoo Lee,*,† Tetsuo Kishi,‡ and Yves Bellouard†

†*Galatea Lab, STI IEM, Ecole Polytechnique Fédérale de Lausanne (EPFL), Rue de la Maladière 71b, Neuchâtel CH-2002, Switzerland*

‡*Department of Chemistry and Materials Science, Tokyo Institute of Technology, 2-12-1 Ookayama Meguro-ku, Tokyo, Japan*

E-mail: seonwoo.lee@epfl.ch

Table of contents

Figure S1. Schematic illustration of the SH imaging technique for fs laser-induced t-Te nanocrystals on tellurite glass.

Figure S2. SH intensity profile perpendicular to the laser writing direction for horizontally, diagonally, and vertically modified lines.

Figure S3. Theoretical calculation of θ dependent polarimetric SH response of t-Te.

Figure S4. Theoretical calculation of φ dependent polarimetric SH response of t-Te.

Figure S5. Cross-polarized images of fs laser-inscribed tracks on tellurite glass.

Figure S6. Effect of writing directions on the SHG-CD of fs-laser induced t-Te.

Figure S7. Effect of writing polarization directions on the SHG-CD of fs-laser induced t-Te.

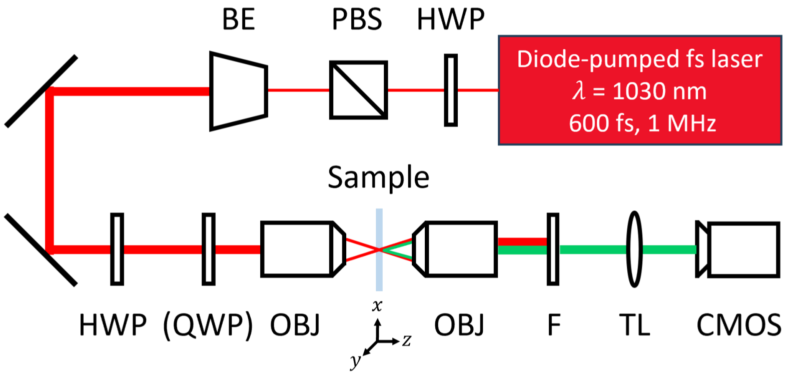

Figure S1. Schematic illustration of the SH imaging technique for fs laser-induced t-Te nanocrystals on tellurite glass. The energy of fs pulse from a diode-pumped laser is attenuated using a polarizing beam splitter (PBS) and a $\lambda/2$ wave plate (HWP). A beam expander (BE) increases the width of the laser beam to fill the entrance pupil of the objective lens (OBJ, NA $= 0.40$). The polarization of the beam is modulated with a $\lambda/2$ wave plate (HWP) or a $\lambda/4$ wave plate (QWP). The beam is loosely focused on a glass specimen using the objective lens. The glass sample is positioned on a 3D high-precision motorized stage. The SH photons (2ω, green line) emitted from the glass are collected by a collecting objective lens. Narrow bandpass SHG filters (F) block the fundamental excitation wavelength (ω, red line). A tube lens (TL) focuses the SH beam on a CMOS image sensor.

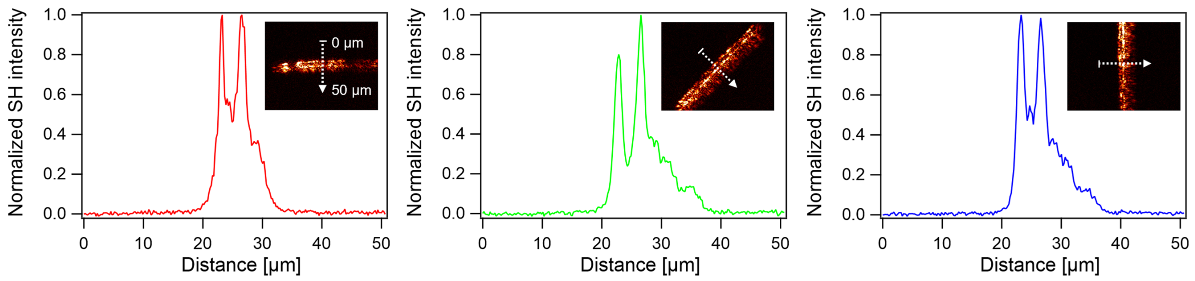

Figure S2. SH intensity profile perpendicular to the laser writing direction for horizontally (red), diagonally (green), and vertically (blue) modified lines. The inset shows SH images corresponding to Figures 2d-f, with white dashed lines indicating the direction of the SH intensity profile.

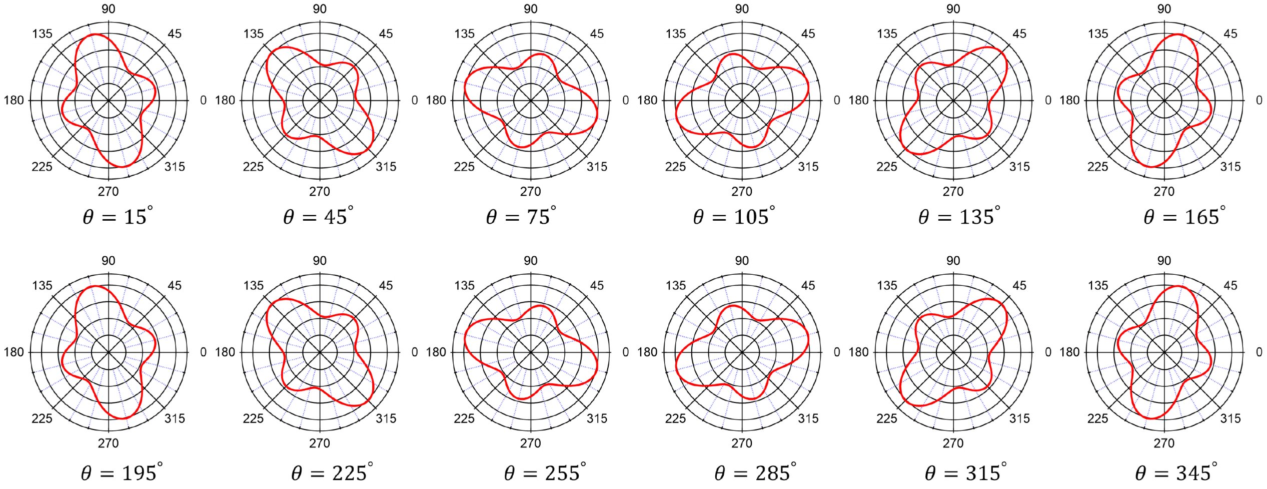

Figure S3. Theoretical calculation of θ dependent polarimetric SH response of t-Te. For fixed angles of $\phi = 10°$, $\varphi = 0°$, SH response is calculated as a function of θ ranging from from 15° to 345° with an interval of 30°. Notably, as θ changes, the cross-shaped polar plot rotates with a periodicity of 180° degree.

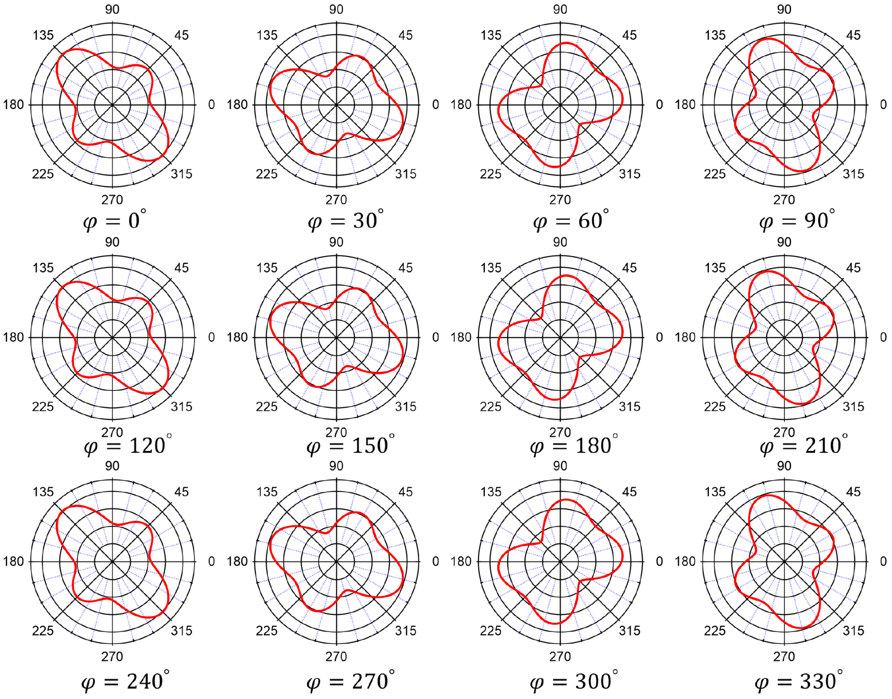

Figure S4. Theoretical calculation of φ dependent polarimetric SH response of t-Te. For fixed angles of $\phi = 10°$, $\theta = 45°$, SH response is simulated as a function of φ ranging from from 0° to 330° with a step of 30°. As φ changes, the cross-shaped polar plot rotates and simultaneously its shape changes. The same shape of plot appears every 120° degree.

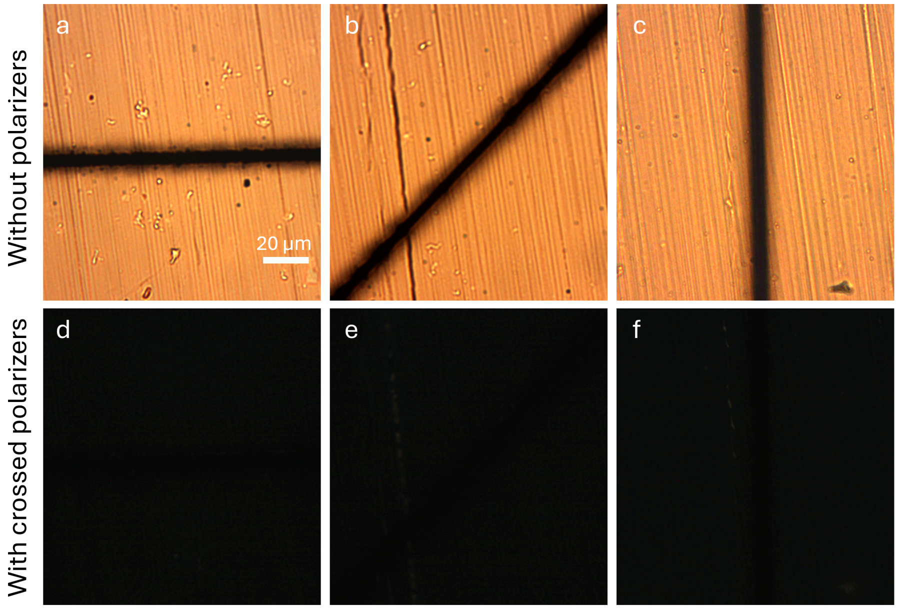

Figure S5. Optical microscope images of the fs laser-inscribed tracks on tellurite glass. The images on the top are taken without polarizers (a-c), while those on the bottom are taken with crossed polarizers (d-f).

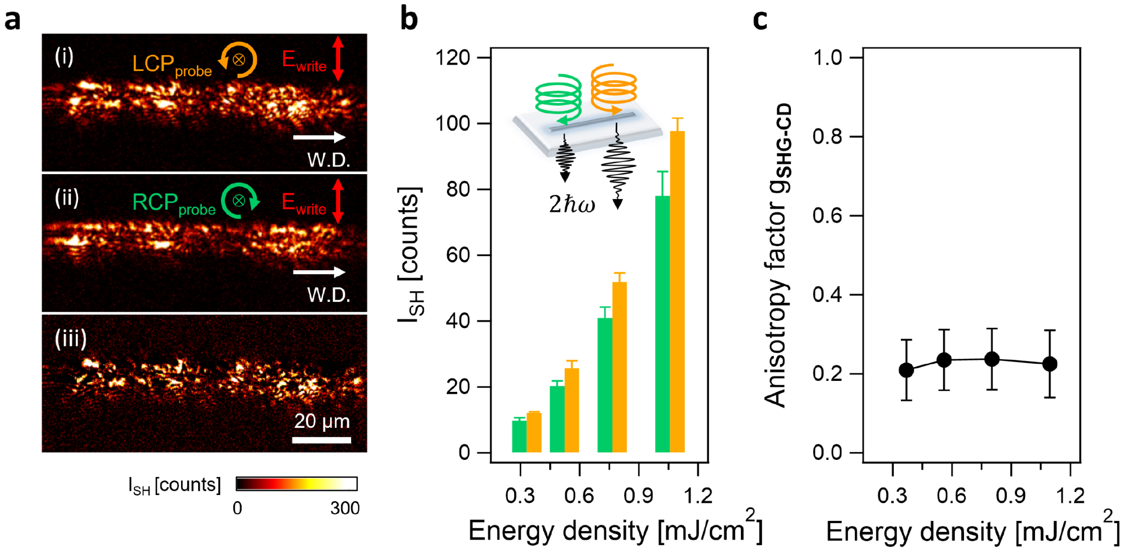

Figure S6. Effect of writing directions on the SHG-CD of fs-laser induced t-Te. (a) SH images of the laser-inscribd lines on the tellurite glass under (i) left-handed circularly polarized (LCP) and (ii) right-handed circularly polarized (RCP) light, together with (iii) the subtracted SH image between (i) and (ii). The laser-inscribed zone is processed using the same experimental condition as in Figure 6a except the writing direction directed from left to right. (b) Averaged SH intensity for the t-Te tracks in Figure S6a under the LCP (orange) and RCP (green) beam as a function of illumination energy density ranging from 0.35 mJ cm^{-2} to 1.10 mJ cm^{-2}. (c) Extracted SHG anisotropy factors g_{SHG-CD} from Figure S6b.

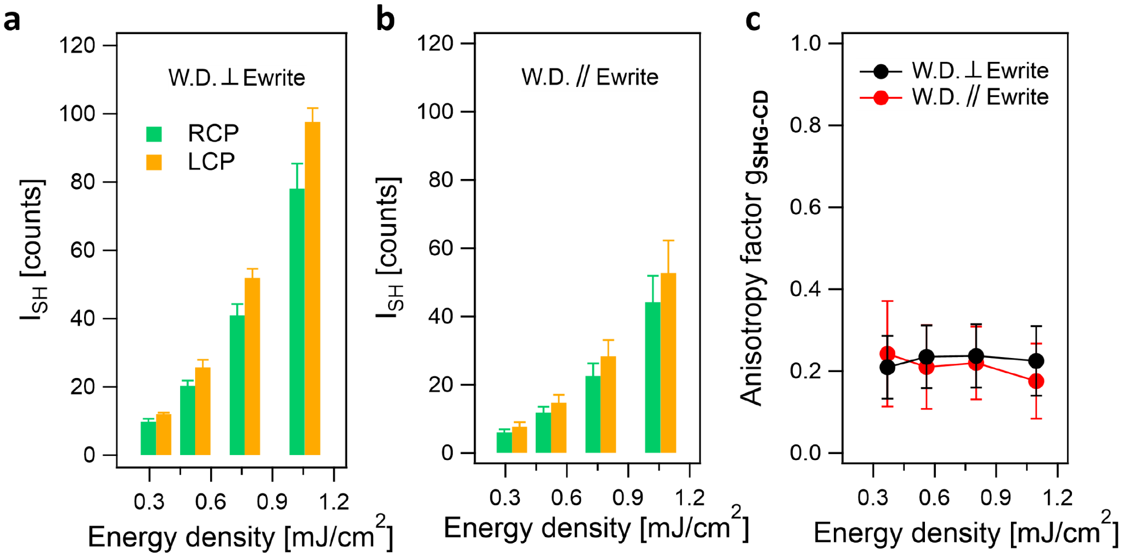

Figure S7. Effect of writing polarization directions on the SHG-CD of fs-laser induced t-Te. Averaged SH intensity for the horizontal t-Te tracks written by (a) transversal (W.D.$\perp E_{\mathrm{write}}$) and (b) longitudinal (W.D.$\| E_{\mathrm{write}}$) polarization, measured with the LCP (orange) and RCP (green) light as a function of its energy density from 0.35 mJ cm^{-2} to 1.10 mJ cm^{-2}. The tracks are written at the repetition rate of 1 MHz, with the pulse energy of 200 nJ, and writing speed of 10 mm s^{-1}. (c) Extracted SHG anisotropy factors g_{SHG-CD} from Figure S7a and S7b.
